# Cbl-b Deficiency in Mice Results in Exacerbation of Acute and Chronic Stages of Allergic Asthma

**DOI:** 10.3389/fimmu.2015.00592

**Published:** 2015-11-20

**Authors:** William F. Carson, Linda A. Guernsey, Anurag Singh, Eric R. Secor, Elizabeth A. Wohlfert, Robert B. Clark, Craig M. Schramm, Steven L. Kunkel, Roger S. Thrall

**Affiliations:** ^1^Department of Pathology, University of Michigan, Ann Arbor, MI, USA; ^2^Department of Immunology, University of Connecticut Health Center, Farmington, CT, USA; ^3^Department of Microbiology and Immunology, University at Buffalo, Buffalo, NY, USA; ^4^Department of Pediatrics, University of Connecticut Health Center, Farmington, CT, USA

**Keywords:** allergic airway disease, Cbl-b, CD4^+^ T cells, inflammation, tolerance

## Abstract

Mice sensitized to ovalbumin (OVA) develop allergic airway disease (AAD) with short-term daily OVA aerosol challenge; inflammation resolves with long-term OVA aerosol exposure, resulting in local inhalational tolerance (LIT). Cbl-b is an E3 ubiquitin ligase involved with CD28 signaling; Cbl-b^−/−^ effector T cells are resistant to regulatory T cell-mediated suppression *in vitro* and *in vivo*. The present study utilized Cbl-b^−/−^ mice to investigate the role of Cbl-b in the development of AAD and LIT. Cbl-b^−/−^ mice exhibited increased airway inflammation during AAD, which failed to resolve with long-term OVA aerosol exposure. Exacerbation of inflammation in Cbl-b^−/−^ mice correlated with increased proinflammatory cytokine levels and expansion of effector T cells in the BAL during AAD, but did not result in either a modulation of lymphocyte subsets in systemic tissues or in OVA-specific IgE in serum. These results implicate a role for Cbl-b in the resolution of allergic airway inflammation.

## Introduction

Mouse models of allergic airway disease (AAD) are important tools for investigating the underlying mechanisms of allergic asthma in humans. In both mice and humans, CD4^+^ T cells are essential for the initiation of airway inflammation in response to allergen exposure. These CD4^+^ T-helper type-2 (T_H_2) cells are responsible for providing the proper signals for B cells to produce immunoglobulin type-E (IgE) antibodies, which are primary mediators of allergic responses through their interactions with receptors on mast cells. Additionally, T_H_2 T cells produce cytokines (e.g., IL-5) that are essential for eosinophil maturation and trafficking to the lung; these cells are hallmarks of allergic inflammation in both mice and humans. Therefore, elucidating the molecular mechanism(s) regulating CD4^+^ T cell activation is an important step toward developing clinical treatments for allergic asthma.

Efficient activation of T cells requires both T-cell receptor (TCR) ligation and costimulation, provided through interactions of T cell surface ligands (e.g., CD28 and ICOS) with corresponding surface receptors on antigen-presenting cells (e.g., B7-1, B7-2, and ICOSL). In the absence of costimulation, TCR ligation results in functional inactivation of T cells, leading to T cell anergy and tolerance induction *in vivo* (1, 2). Therefore, the interactions of numerous costimulatory pathways with TCR stimulation are essential for activation of T cells during an inflammatory response.

Negative regulators of costimulatory signal transduction proteins play an important role in T cell development and activation *in vivo*. One such regulator is the Casitas B cell lymphoma (Cbl) family of E3 ubiquitin ligases, which play an integral role in mediating TCR signal strength and subsequent survival and activation (3). Two family members, c-Cbl and Cbl-b, regulate TCR signal strength during positive and negative selection in the thymus, and in activation and survival in the periphery (4). Specifically, Cbl-b is important for mediating CD28 signal strength during TCR ligation, through negative control of the signal transduction proteins Vav1, PKCθ, and PLCγ (5–8). T cells lacking Cbl-b exhibit a decreased threshold of activation resulting in increased proliferative ability and increased IL-2 production *in vitro* (9). Cbl-b plays an integral role in governing anergy induction in NK (10) and NK-T cells (11), and inhibition of Cbl-b expression or function in these cells can serve to enhance their anti-tumor activities. Cbl-b also negatively regulates B cell (12) and mast cell function (13). Mice deficient in Cbl-b develop spontaneous autoimmunity at advanced age, characterized by autoantibody production and infiltration of non-lymphoid organs by activated T and B cells (14); also, they have an increased propensity to develop experimentally induced autoimmunity [e.g., experimental autoimmune encephalomyelitis (5)]. Additionally, Cbl-b T cells are resistant to regulatory T cell (T_reg_)-mediated suppression both *in vivo* and *in vitro*, as well as resistant to the immunosuppressive properties of transforming growth factor-beta (TGF-β) (15, 16).

Ovalbumin (OVA)-induced AAD in mice generates a biphasic response whereby short-term aerosol challenge results in AAD, while long-term aerosol challenge results in a resolution of airway inflammation, referred to as local inhalational tolerance (LIT) (17). The induction of airway inflammation in response to OVA aerosol is critically dependent on T_H_2 cells (18). LIT may be mediated by T_regs_, as cells with this phenotype are enriched in local lung compartments at timepoints correlating with the resolution of inflammation (19). As Cbl-b^−/−^ T cells have been shown to exhibit a decreased activation threshold, and are resistant to Treg-mediated suppression (15, 16), these mice provide a unique environment to study the role of CD4^+^ T cell–Treg interactions. A recent study has identified an important role for Cbl-b in mediating allergic inflammation, with Cbl-b^−/−^ mice on a BALB/c background exhibiting enhanced T_H_2 and T_H_9 responses to OVA-induced AAD (20). This study implicates an important mechanistic role for Cbl-b in governing helper T cell responses to allergen through regulation of STAT6 and subsequent IL-4 production; however, these studies did not directly address the effect of Cbl-b deficiency on T cell–T_reg_ interactions in the context of allergic airway inflammation and the induction of LIT following chronic allergen exposure. Therefore, the purpose of this study was to determine if Cbl-b deficiency (Cbl-b^−/−^ mice) had an effect the development of either the acute (inflammatory) stage or the chronic (resolution) stage of this OVA-induced model, and to determine if the modulated inflammatory response correlated with changes in T_reg_ numbers or functions.

## Materials and Methods

### Animals

Female C57BL/6 mice, 3–4 months of age and weighing 15–20 g, were purchased from the Jackson Laboratory (Bar Harbor, ME, USA) and housed conventionally in plastic cages with corncob bedding. Cbl-b^−/−^ mice harboring a global homozygous deletion of the Cbl-b gene were bred in the laboratory of Robert Clark, M.D., from breeding pairs originally obtained with permission from Hua Gu, Ph.D. (Columbia University College of Physicians and Surgeons, New York, NY, USA) (5). The animal room was maintained at 22–24°C with a daily light/dark cycle. Chow and water were supplied *ad libitum*. The protocols for animal use were approved by the Animal Care Committee at the University of Connecticut Health Center.

### Ovalbumin Exposure Protocol

As previously described (17, 21), mice were immunized with three weekly intraperitoneal (i.p.) injections of a suspension containing 25 μg of OVA (grade V, Sigma Chemical, St. Louis, MO, USA) and 2 mg of aluminum hydroxide (alum) in 0.5 ml of 0.9% sodium chloride in sterile H_2_O (saline). One week after the last injection, the mice were exposed to 1% aerosolized OVA in 0.9% sodium chloride in sterile H_2_O (saline) for 1 h/day for either 7 (AAD) or 42 days (LIT). The aerosols were generated by a BANG nebulizer (CH Technologies, Westwood, NJ, USA) into a 7.6 l nose-only inhalation exposure chamber to which individual restraint tubes were attached. Chamber airflow was 6 l/min, and aerosol particle size of OVA was monitored by gravimetric analysis with a Mercer cascade impactor (In-Tox Products, Moriarty, NM, USA). The mass median aerodynamic diameter and geometric SD were 1.4 and 1.6 μm, respectively. The estimated daily inhaled OVA dose ~30–40 μg/mouse. Twenty-four hours after the final aerosol exposure, the mice were killed by ketamine/xylazine overdose and subsequent exsanguination.

### Bronchoalveolar Lavage/Tissue Analysis

At sacrifice, local lung compartments such as bronchoalveolar lavage (BAL) fluid, lung tissue, and hilar lymph node (HLN) and systemic compartments such as inguinal lymph node (ILN) and spleens were harvested and processed for the isolation and enumeration of leukocytes. For collection of BAL, lungs were lavaged *in situ* with five 1 ml aliquots of 0.9% sodium chloride in sterile H_2_O (saline). The BAL fluid of wild-type and Cbl-b^−/−^ mice at naïve, AAD and LIT timepoints were centrifuged, the cellular pellet washed, and total viable white blood cells (WBCs) were enumerated using a hemacytometer and vital dye exclusion (nigrosin) as a measure of viability. Cytospin preparations of BAL fluid were stained with May-Grunwald and Giemsa for cell differential analysis. Total eosinophils in BAL fluid were calculated by multiplying the percentages obtained by differential cell analysis by the total WBC count.

For the isolation of cells from lung tissue, mice were perfused with PBS/Heparin prior to dissection. Lungs were removed, minced, digested in collagenase (150 U/ml) (Invitrogen, Carlsbad, CA, USA), and the cellular suspension separated on a Percoll (Sigma Chemical, St. Louis, MO, USA) density gradient for the isolation of lymphocytes. Lymph nodes and spleens were harvested and mechanically disrupted into a single-cell suspension. Hypotonic lysis with ammonium chloride lysis buffer (150 mM NH_4_Cl, 10 mM NaHCO_3_, 0.1 mM disodium EDTA in sterile H_2_O) was used to eliminate erythrocytes. For all tissue samples, total WBCs were obtained using a hemocytometer with nigrosin dye exclusion as a measure of viability.

### Flow Cytometry and Immunofluorescence

Cells isolated from the BAL and other tissues were analyzed via flow cytometry using the following monoclonal antibodies: anti-CD8a-FITC (53-6.7), anti-CD25-PE (PC61), anti-CD4-PeCy7 (RM4-5), anti-CD3e-PerCP-Cy5.5. (145-2C11), and anti-CD19-PerCP-Cy5.5 (1D3) (BD PharMingen, San Diego, CA, USA). Samples were washed in PBS containing 0.2% bovine serum albumin and 0.1% NaN_3_. Aliquots containing 10^4^–10^6^ cells were incubated with 100 μl of appropriately diluted antibodies for 30 min at 4°C. After staining, the cells were washed with the above PBS solution, and relative fluorescence intensities were determined on a four-decade log scale by flow cytometric analysis using a LSR II (Becton-Dickinson, San Diego, CA, USA). For the identification of T_reg_, intracellular staining of Foxp3 protein was used. Briefly, cells stained with the antibodies mentioned previously (i.e., anti-CD3e, anti-CD4, and anti-CD25) were permeabilized using fixation/permeabilization buffer following the manufacturer’s protocol, and stained using either anti-Foxp3-FITC (FJK-16s) or anti-Foxp3-APC (FJK16s) with corresponding isotype controls, IgG2a-FITC or -APC (eBioscience, San Diego, CA, USA).

### Histology

After sacrifice, unmanipulated (not subject to BAL) and non-inflated lungs from separate animals were removed and fixed with 10% formalin. Preserved lungs were embedded in paraffin and tissue sections were prepared using a microtome and mounted onto glass slides. Mounted tissue sections were then stained with hematoxylin and eosin for standard evaluation, and periodic acid-Schiff (PAS) for evaluation of mucus production.

### IgE ELISA

Ovalbumin-specific IgE levels were measured by ELISA using isotype-specific capture monoclonal antibodies following a standard protocol (22). Briefly, IgE was captured from diluted serum using Immulon 2 microtiter plates (Dynatech Laboratories, Chantilly, VA, USA) coated with anti-mouse IgE (R35-72; BD PharMingen, San Diego, CA, USA) at 2 μg/ml in 0.1 mol/l carbonate, pH 9.5. Detection was with an OVA-digoxigenin conjugate followed by horseradish peroxidase-conjugated anti-digoxigenin. Plates were developed with the TMB microwell peroxidase substrate system (Kirkegaard and Perry Laboratories, Gaithersburg, MD, USA).

### Multiplex Cytokine Analysis

Concentrations of proinflammmatory cytokines in BAL fluid were measured using a mouse cytokine custom bead assay (Bio-Rad, Hercules, CA, USA) and analyzed using a Luminex Bio-Plex 200 system (Bio-Rad), according to the manufacturer’s protocol. Briefly, the 96-well multiplex assay plate was coated with anti–mouse cytokine conjugated beads for the capture of IL-2, IL-4, IL-5, IL-6, IL-10, IL-12p70, IL-13, IFN-γ, and CCL5 (Eoxtaxin). Plates were rinsed two times with the provided wash buffer, standards and experimental samples were loaded, and the plate was incubated for 30 min at room temperature with gentle vortex. After three washings, the biotinylated mouse cytokine detection antibody was added for 30 min at room temperature with gentle vortex. The plates were washed again, and PE-conjugated streptavidin was added for 10 min at room temperature with gentle vortex. Plates were washed and read using the Luminex Bio-Plex 200 system plate reader according to the manufacturer’s instructions. Standard curves were generated by analysis of serially diluted samples of a known cytokine standard provided by the manufacturer. The threshold of detection of each cytokine is 5 pg/ml.

### Statistical Analysis

Analysis of variance (ANOVA) followed by *post hoc* two-tailed Student’s unpaired *t*-tests were used for data analysis. All statistical analysis was performed using either the JMP IN 5.1 statistical software package (SAS Institute, Cary, NC, USA) or the GraphPad Prism 4 software package (GraphPad Software, La Jolla, CA, USA).

## Results

### Cbl-b^−/−^ Mice Exhibit Increased BAL WBCs and Eosinophils During AAD and LIT

**Figure 1:**
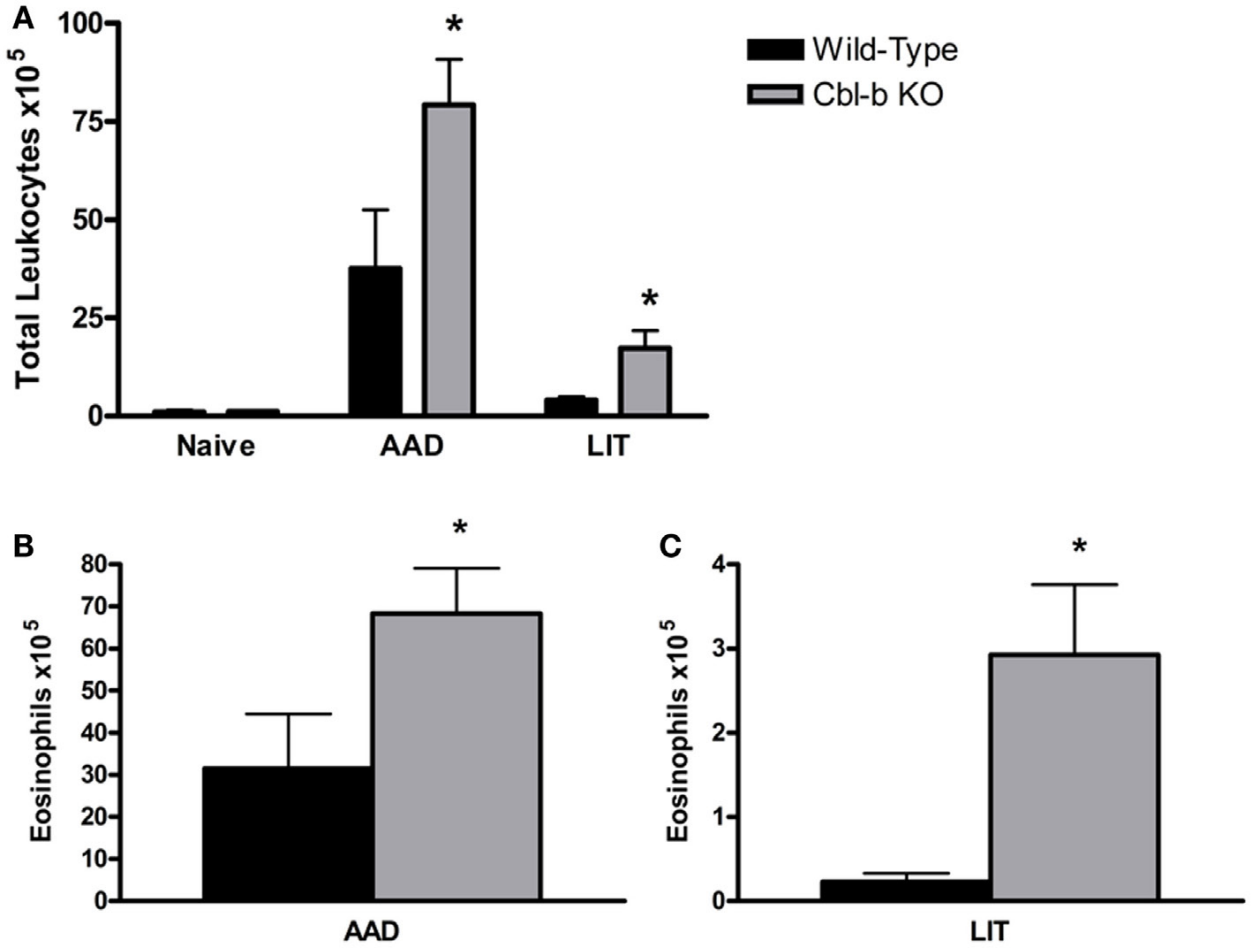
**Total white blood cells and eosinophils are increased in the BAL of Cbl-b^−/−^ mice at AAD and LIT**. **(A)** Total white blood cells (WBCs) in BAL fluid were enumerated using a hemacytometer and nigrosin stain for viability. **(B,C)** Cytokine preparations of BAL fluid from AAD **(B)** and LIT **(C)** mice were stained with May-Grunwald and Giemsa for cell differential analysis. Total eosinophils in BAL fluid were calculated by multiplying the percentages obtained by differential cell analysis by the total WBC count. Data represent the mean + SEM of 7–9 mice per group. *p < 0.05 vs. respective AAD or LIT wild-type mice.

Prior to sensitization and aerosol challenge, Cbl-b^−/−^ mice had similar BAL cell composition as wild-type naive controls, consisting primarily of macrophages (98.3 ± 0.5% WT and 96.1 ± 0.9% Cbl-b^−/−^). At AAD, Cbl-b^−/−^ mice developed enhanced airway inflammation compared to wild-type controls, with a significant increase in total BAL leukocytes (*p* = 0.0016; Figure 1A) consisting primarily of eosinophils (83.2 ± 1.6% WT and 82.1 ± 2.2% Cbl-b^−/−^). The total number of eosinophils in Cbl-b^−/−^ BAL fluid at AAD was increased over twofold as compared to wild-type mice (*p* = 0.002; Figure 1B). At LIT, while both wild-type and Cbl-b^−/−^ mice exhibited significant decreases in total BAL WBCs from their respective AAD levels (*p* < 0.05 for LIT vs. AAD) (Figure 1A), there remained a significant increase in total WBCs (over 4-fold; *p* = 0.046; Figure 1A) and eosinophils (over 12-fold; *p* = 0.030; Figures 1B,C) in the BAL of Cbl-b^−/−^ mice as compared to wild-type mice.

### Cbl-b^−/−^ Mice Demonstrated Histological Evidence of Increased Lung Inflammation and Mucus Production During AAD and LIT

**Figure 2:**
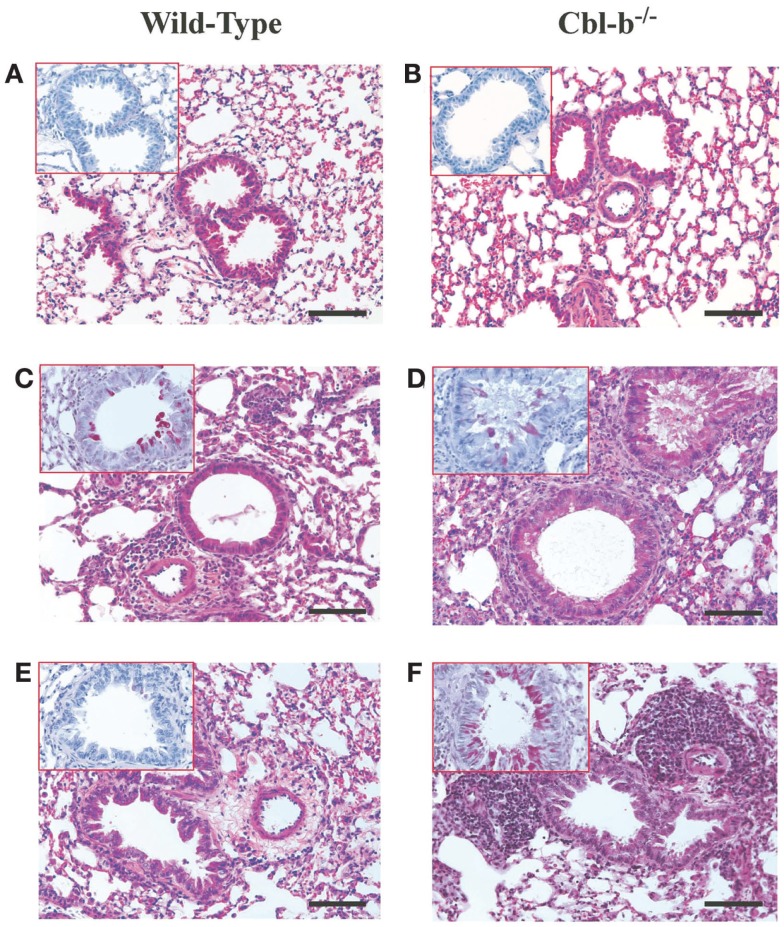
**Lung histology from wild-type and Cbl-b mice**. Formalin-fixed lungs from wild-type or Cbl-b^−/−^ mice at naïve **(A,B)**, AAD **(C,D)** or LIT **(E,F)** were stained with Hematoxin and Eosin (H&E) for analysis of tissue inflammation, and periodic acid-Schiff (PAS) (inserts) for the identification of mucus production. All sections at 20× magnification. Scale bar = 100 μm.

Cbl-b^−/−^ mice have an increased propensity to develop both spontaneous autoimmunity and experimentally induced autoimmune diseases (5, 14). To ensure that the lung histological observations seen in Cbl-b^−/−^ mice were not due to pre-existing airway inflammation prior to sensitization and aerosol challenge, lungs from age-matched and older (18 weeks) Cbl-b^−/−^ mice were analyzed for airway inflammation. Naïve wild-type (Figure 2A), naïve aged-matched Cbl-b^−/−^ (Figure 2B), and 18-week-old Cbl-b^−/−^ lungs (data not shown) were comparable with little to no evidence of airway inflammation prior to sensitization and aerosol challenge.

At AAD, qualitative analysis of lungs from Cbl-b^−/−^ mice showed increased perivascular and peribronchial inflammation (Figure 2D) as compared to wild-type mice (Figure 2C). Similar increases in mucus production (Figure 2D – insert) were seen for Cbl-b^−/−^ mice as compared to wild-type mice (Figure 2C – insert), with significant mucus plugging apparent in the bronchial airways. This increase in lung inflammation was striking at LIT, with significant perivascular and peribronchial inflammation persisting in Cbl-b^−/−^ mice (Figure 2F) as compared to the resolution of inflammation in wild-type mice (Figure 2E) in response to long-term aerosol challenge. Along with increased inflammation, Cbl-b^−/−^ mice also showed evidence of mucus production in the airways at LIT (Figure 2F – insert), whereas wild-type mice had airways that were clear of mucus production (Figure 2E insert).

### Exacerbation of Inflammation in Cbl-b^−/−^ Mice Resulted in Modulation of the Lymphocyte Populations in the HLN at LIT but Not AAD

As Cbl-b mice exhibited increased airway inflammation and eosinophilia in response to aerosol challenge, we sought to determine if the total number and/or frequency of lymphocyte subsets in local (HLN) and systemic (ILN) tissues were affected as well. No significant differences were seen in percentages or total numbers of T or B cell subsets analyzed in the wild-type or Cbl-b^−/−^ mice at naïve or sensitized timepoints (data not shown). At AAD, a similar pattern was observed, with no significant differences in T or B cell subsets in either local lung or systemic tissues, either in regards to cell percentages (data not shown) or total cells (Table 1A). In a similar manner, no significant differences were found between percentages or total numbers of CD8^+^, CD4^+^, or CD19^+^ lymphocytes in the BAL, ILN, and spleen at LIT (Table 1B). However, in the HLN at LIT, Cbl-b^−/−^ mice had increased numbers of all lymphocytes analyzed as compared to wild-type mice, indicative of an increase in total cellularity of the node. This increase was not due to changes in the distribution of lymphocyte subsets in the HLN, as percentages of CD8^+^, CD4^+^, and CD19^+^ cells were equivalent (data not shown).

**Table 1:** **Lymphocyte numbers in local and systemic tissues at AAD and LIT**.

		CD8^+^	CD4^+^	CD19^+^
**(A) AAD**				
BAL	Wild-type	6.2 ± 2	13.8 ± 5	11.7 ± 9
	Cbl-b^−/−^	15.8 ± 4	23.9 ± 6	10.1 ± 4
HLN	Wild-type	61.8 ± 25	79.9 ± 34	159.3 ± 128
	Cbl-b^−/−^	143.3 ± 43	160.2 ± 48	424.7 ± 163
ILN	Wild-type	24.2 ± 9	27.9 ± 10	20.4 ± 6
	Cbl-b^−/−^	25.8 ± 7	23.9 ± 6	21.6 ± 7
Spleen	Wild-type	171.7 ± 28	303.2 ± 65	169.5 ± 34
	Cbl-b^−/−^	172.5 ± 39	326.3 ± 68	148.3 ± 20
**(B) LIT**				
BAL	Wild-type	0.9 ± 0.2	1.4 ± 0.2	0.5 ± 0.04
	Cbl-b^−/−^	9.8 ± 5	7.6 ± 4	3.3 ± 1
HLN	Wild-type	116.7 ± 37	111.4 ± 28	64.6 ± 1
	Cbl-b^−/−^	291.1 ± 65*	259.9 ± 53*	265.0 ± 43*
ILN	Wild-type	41.8 ± 10	44.8 ± 10	7.1 ± 1
	Cbl-b^−/−^	21.1 ± 7	19.7 ± 6	3.2 ± 2
Spleen	Wild-type	142.3 ± 53	230.3 ± 74	159.6 ± 74
	Cbl-b^−/−^	171.3 ± 38	232.3 ± 48	310.3 ± 125

*Single-cell suspensions of the indicated tissues and tissue compartments were analyzed via flow cytometry for the presence of CD8^+^, CD4^+^, and CD19^+^ lymphocytes. Total numbers (cells × 10^4^) were obtained as indicated in the Section “Materials and Methods.” *n* = 5–8 mice per group*.

*p < 0.05 vs. wild-type.

During the progression from AAD to LIT in this OVA model, the ratio of T cell subsets in the BAL of wild-type mice shows a progression from a CD4^+^ environment (~2:1) to a normalized CD4:CD8 environment (~1:1) (21). In Cbl-b^−/−^ mice, the ratio of total CD4:CD8 T cells in the BAL was skewed toward CD8^+^ T cells (~1.5) in comparison to the ratio in wild-type mice (~2.2). However, this difference was not apparent for total numbers of CD4^+^ or CD8^+^ T cells in the BAL when compared between wild-type and Cbl-b^−/−^ mice. At LIT, the BAL of Cbl-b^−/−^ mice showed a skewed CD4:CD8 T cell ratio (~0.8) as compared to wild-type mice (~1.5), which was largely due to a statistically significant increase in percentages of CD8^+^ T cells (of lymphocytes, 10.3 ± 2.8% for wild-type vs. 23.6 ± 3.0% for Cbl-b^−/−^, *p* = 0.019). While all lymphocyte subsets analyzed were increased in terms of total number in the Cbl-b^−/−^ BAL, these differences were not statistically significant, primarily due to increased variation in total numbers of lymphocytes in the Cbl-b^−/−^ mice.

### Cbl-b Deficiency Did Not Result in Increased OVA-Specific IgE Production in Serum

**Figure 3:**
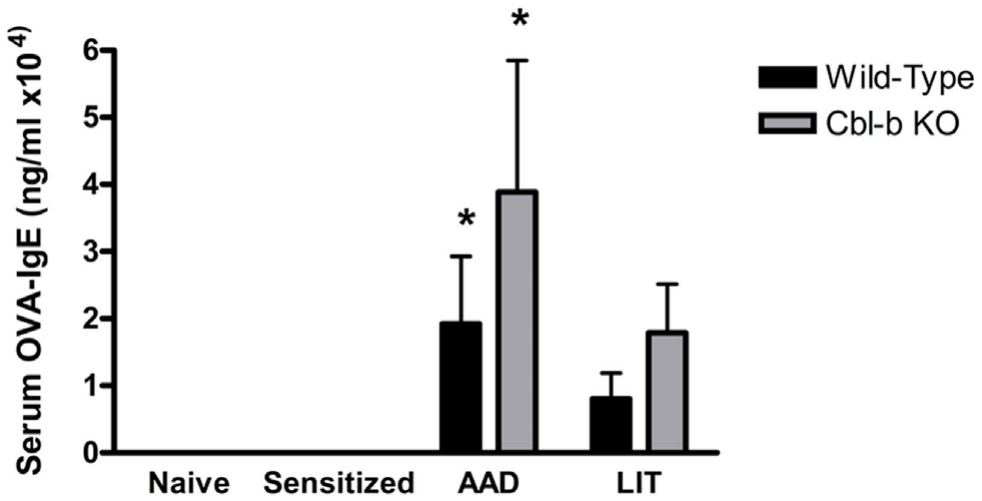
**Systemic serum OVA-IgE levels in wild-type and Cbl-b mice are increased at AAD**. Serum from wild-type or Cbl-b mice was isolated at the indicated timepoints and analyzed via OVA-specific IgE ELISA. Data represent the mean ± SEM of 6–8 mice per group. *p < 0.05 vs. Naïve/Sensitized levels.

A standard outcome of this OVA-induced model of AAD is the production of OVA-specific IgE in the serum, which is a hallmark of T_H_2 allergic responses in both mice and humans. Serum levels of OVA-IgE in naïve or sensitized wild-type and Cbl-b^−/−^ mice were minimal and showed no significant differences between strains (Figure 3). At AAD, both wild-type and Cbl-b^−/−^ exhibited elevated serum OVA-IgE levels; however, there were no significant differences observed in OVA-IgE concentrations between mouse strains (*p* = 0.38; Figure 3). Similarly, OVA-IgE levels remained elevated in both mouse strains at LIT; however, there were no significant differences observed between wild-type and Cbl-b^−/−^ mice (*p* = 0.20; Figure 3).

### Cbl-b^−/−^ Mice Exhibited Increased Levels of Proinflammatory Cytokines and Chemokines in the BAL During AAD and LIT

**Figure 4:**
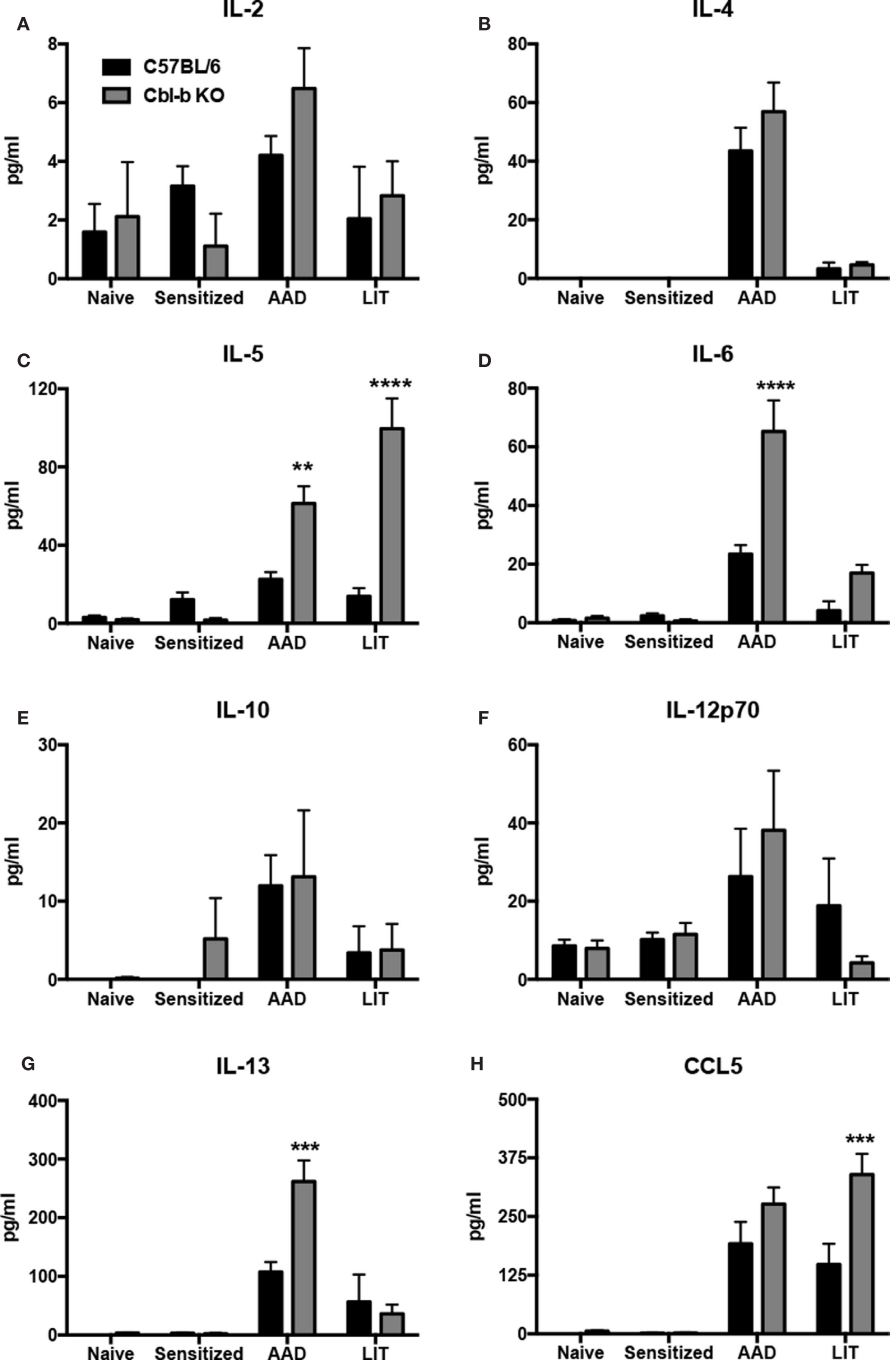
**Proinflammatory cytokines and chemokines were increased in the BAL of Cbl-b^−/−^ mice at AAD and LIT**. Concentrated BAL samples from wild-type (C57BL/6) and Cbl-b^−/−^ mice at the indicated timepoints were analyzed via multiplex bead assay for the presence of IL-2 **(A)**, -4 **(B)**, -5 **(C)**, -6 **(D)**, -10 **(E)**, -12p70 **(F)**, and -13 **(G)** along with the T cell chemotactic factor CCL5 **(H)**. Data represent the mean ± SEM of 4–6 mice per group. **p < 0.01, ***p < 0.001, ****p < 0.0001 vs. wild-type.

As the increased airway inflammation in Cbl-b^−/−^ mice during AAD and LIT did not correlate with modulations in lymphocyte populations in the BAL (Table 1) or increases in systemic OVA-specific IgE (Figure 3), we sought to determine if increases in proinflammatory cytokines in the local lung environment correlated with the exacerbation of inflammation in Cbl-b^−/−^ mice. BAL proteins from both wild-type and Cbl-b^−/−^ mice at the indicated timepoints were analyzed via multiplex bead assay for the presence of numerous proinflammatory cytokines, along with the T cell chemotactic factor CCL5 (RANTES) (Figure 4).

No significant differences were observed between wild-type and Cbl-b^−/−^ mice at the indicated timepoints for levels of IL-2 (Figure 4A), IL-4 (Figure 4B), IL-10 (Figure 4E), or IL-12p70 (Figure 4F). However, Cbl-b^−/−^ mice had increased levels of the T_H_2 cytokines IL-5 (Figure 4C) and IL-13 (Figure 4G), along with the proinflammatory cytokine IL-6 (Figure 4D) in the BAL at AAD as compared to wild-type. At LIT, Cbl-b^−/−^ mice had increased levels of both IL-5 (Figure 4C) and CCL5 (Figure 4H) in the BAL as compared to wild-type, while levels of IL-6 and IL-13 returned to levels comparable to wild-type. In addition, no significant differences were observed in IFNγ levels between wild-type and Cbl-b^−/−^ mice, as IFNγ was not detected in the BAL at any timepoint of the model in either mouse strain (data not shown).

### CD4^+^ CD25^+^ Foxp3^−^ Activated T Cells Are Increased in the BAL of Cbl-b^−/−^ Mice at AAD

**Figure 5:**
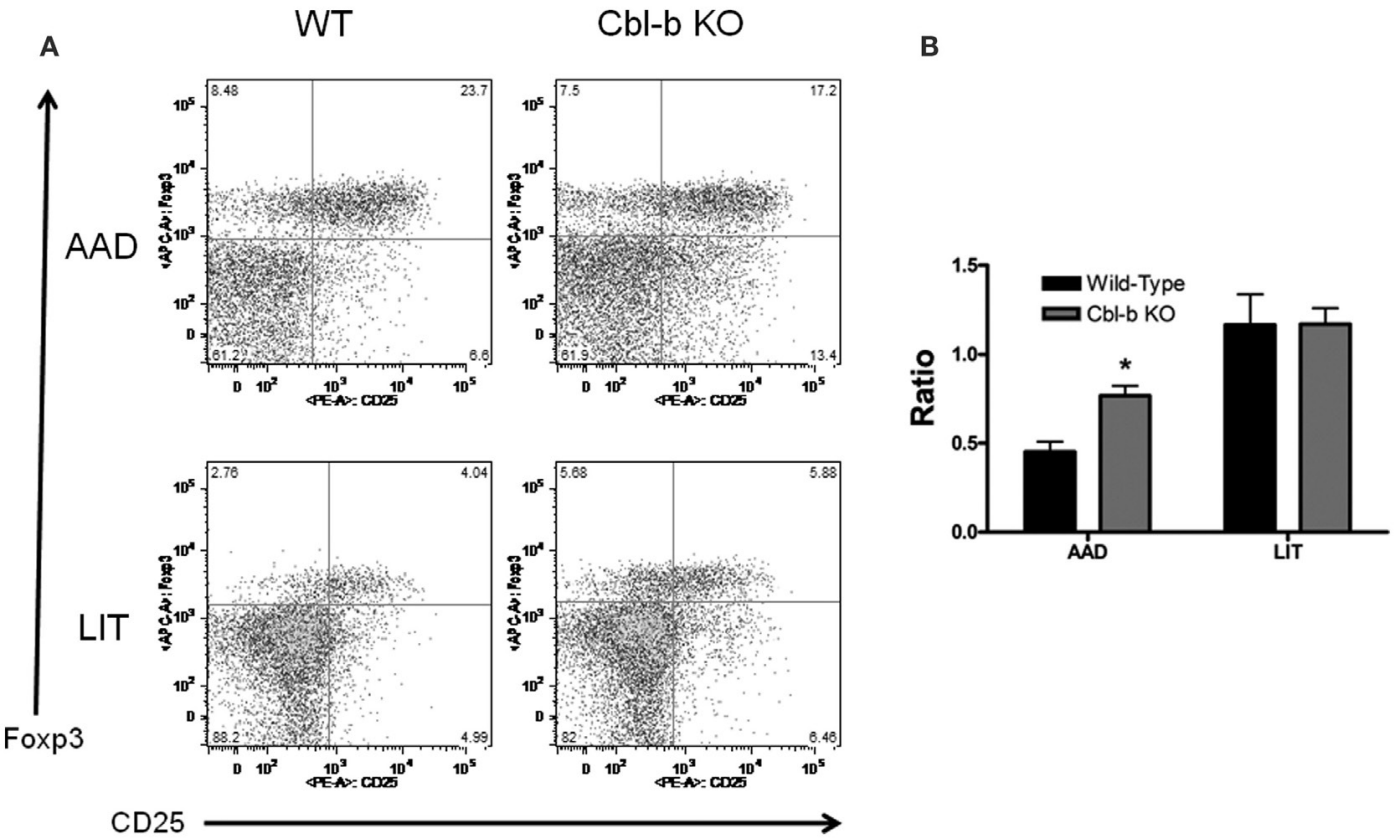
**Percentage of CD4^+^ CD25^+^ Foxp3^−^ T cells are increased in BAL of Cbl-b^−/−^ mice during AAD**. **(A)** Representative flow cytometric analysis of BAL fluid from wild-type and Cbl-b^−/−^ mice at AAD and LIT. Samples were gated on CD3^+^ CD4^+^ T cells. **(B)** Ratio of BAL T cells at AAD and LIT in wild-type and Cbl-b^−/−^ mice. Ratios were obtained by dividing the percentage of CD4^+^ CD25^+^ Foxp3^−^ T cells by the percentage of CD4^+^ CD25^+^ Foxp3^+^ T cells. Data represent the mean ± SEM of 5 mice per group. *p < 0.05 vs. wild-type.

To further characterize the CD4^+^ T cell response in local lung vs. systemic tissues during AAD and LIT, CD4^+^ T cells were analyzed for expression of the activation marker CD25 and the regulatory T cell-specific transcription factor Foxp3. As with the previous lymphocyte characterization, no significant differences were found between wild-type and Cbl-b^−/−^ mice for percentages of CD4^+^ CD25^+^ Foxp3^−^ and CD4^+^ CD25^+^ Foxp3^+^ in systemic compartments at all timepoints (data not shown). However, Cbl-b^−/−^ mice did exhibit an increase in the Foxp3^−^ population in the BAL during AAD, in terms of percentage of the total CD4^+^ population (8.64 ± 0.8% for wild-type vs. 12.6 ± 0.9% for Cbl-b^−/−^, *n* = 5 for each group, *p* < 0.01) (Figure 5A). When these values are expressed as a ratio, the differences in cell composition during AAD become apparent, with an increase in Foxp3^−^ in Cbl-b^−/−^ mice (Figure 4B). At LIT, this difference was no longer apparent, as percentages of Foxp3^−^ and Foxp3^+^ were equivalent between wild-type and Cbl-b^−/−^ mice (4.90 ± 0.9% for wild-type vs. 5.86 ± 0.8% for Cbl-b, *n* = 5 for each group) (Figure 5B).

## Discussion

The role of Cbl-b in the regulation of peripheral T cell responses is well documented; however, its role in mediating T_H_2-type allergic responses remains elusive. The results of these studies indicate that Cbl-b plays an integral role in regulating the intensity of an allergic airway response in mice, as Cbl-b^−/−^ mice exhibit increased inflammation during acute and chronic stages of an OVA-induced AAD model. This exacerbation does not appear to be due to the inherent autoimmune phenotype of Cbl-b^−/−^ mice (9), since age-matched (i.e., 18-week) naïve Cbl-b^−/−^ mice did not develop airway inflammation. These results indicate that Cbl-b deficiency can influence the severity of AAD through the dysregulation of T cell responses.

Long-term aerosol exposure in OVA-sensitized mice results in the resolution of AAD, characterized by a reversal of eosinophilia and mucus secretion in the airways (17, 21). Interestingly, the resolution of AAD was inhibited in Cbl-b^−/−^ mice, as these mice exhibited persistent eosinophilia and mucus secretion in the airways at the chronic stage as compared to wild-type controls. Previous reports have indicated that the resolution of AAD in wild-type mice is dependent on continuous aerosol exposure (17). These results suggest that the inability of Cbl-b^−/−^ mice to resolve AAD in response to long-term continuous aerosol exposure may be due to its inability to mount a T_reg_-mediated local inhalational tolerant state, perhaps via the dysfunctional nature of T_reg_ interactions in these mice.

Along with histological evidence of increased airway inflammation, Cbl-b^−/−^ mice exhibited increased levels of the proinflammatory cytokines IL-5, IL-6, and IL-13 in the BAL at AAD. Additionally, levels of the T_H_2 cytokine IL-5 and the T cell chemokine CCL5 were increased at LIT above wild-type levels, indicating a role for these immune modulating signaling proteins in the exacerbation of inflammation during prolonged aerosol exposure in Cbl-b^−/−^ mice. IL-5 and IL-13 are T_H_2 cytokines associated with the development of airway inflammation in mice (23) and humans (24, 25); the observed increase in these proinflammatory cytokines in Cbl-b^−/−^ mice is suggestive of exacerbated T_H_2 cell responses to aerosol challenge during acute inflammation. While the role of IL-6 in allergic inflammation is less clear, it is a potent inflammatory cytokine, and has been shown to play a role in fungal-induced asthma (26). Additionally, IL-6 has been shown to act as a negative regulatory of T_reg_ function (27, 28), suggesting that the increase in IL-6 at AAD in Cbl-b^−/−^ may be evidence of “resistance to regulation” during acute inflammation through IL-6 mediated inhibition of T_reg_ suppression. At LIT, levels of IL-5 were significantly increased compared to wild-type, which may provide a mechanism for the increased eosinophils in the BAL of Cbl-b^−/−^ mice at LIT, as IL-5 is a potent chemoattractant and activation factor for eosinophils (29, 30). Interestingly, levels of CCL5 in the BAL of Cbl-b^−/−^ mice were increased above wild-type levels only at LIT, suggesting that increased T cell chemokine expression in Cbl-b^−/−^ mice may play a role at exacerbating inflammation at LIT.

Cbl-b^−/−^ effector T cells (T_eff_) have been shown to be “resistant to regulation,” in that they are unresponsive to the suppressive effects of either T_reg_ or the immunosuppressive cytokine TGF-β both *in vitro* and *in vivo* (15, 16). This deficiency in regulation is not due to intrinsic defects in Cbl-b^−/−^ T_reg_, as these cells are capable of suppressing wild-type T_eff_ proliferation *in vitro* (15). However, the ability of naïve Cbl-b^−/−^ T_eff_ to respond to T_reg_*in vivo* and *in vitro* is severely impaired (15), suggesting that “resistance to regulation” may be due to defects in the cross-talk necessary between T_eff_ and T_reg_ for the suppression of inflammation. T_reg_ are believed to play an important role in the development and/or maintenance of LIT, as Foxp3^+^ T cells are increased in local lung compartments in response to long-term aerosol challenge (19). Therefore, increased inflammation in Cbl-b^−/−^ mice may be due to defects in regulation of activated T cell-mediated inflammation by T_reg_ during the progression from AAD to LIT. Importantly, the definition of activated T cell in this model system does not address the issue of antigen specificity. Further studies are required to determine if the increase in inflammation observed in Cbl-b^−/−^ are due to an expansion of OVA-specific T cell subsets, enhanced activation of an equivalent population of OVA-specific T cells, or some combination of both phenomena.

Cbl-b plays an important role in T cell activation through its interaction with signal transduction factors downstream of CD28. Additionally, Cbl-b plays a role in regulating T_eff_ function after antigen-induced activation through involvement with cell-intrinsic and cell-to-cell regulatory pathways. In terms of cell-intrinsic pathways, Cbl-b has been shown to play an important role in anergy induction in response to tolerizing signals (31, 32). For example, T cells from Cbl-b^−/−^ mice exhibit an inability to develop anergy in response to calcium ionophore treatment *in vitro*, and *in vivo* antigen challenge of *in vitro* anergized Cbl-b^−/−^ T cells with cognate antigen results in T cell-mediated lethality in recipient mice (33). Calcium-mediated anergy induction proceeding through calcineurin has also been shown to rely on Cbl-b, along with other E3 ubiquitin ligases, as expression of Cbl-b was increased in sustained calcium flux-mediated anergic T cells (7). Based on these findings, it is possible that the increased airway inflammation in Cbl-b^−/−^ mice may be due to a deficiency in anergy induction in activated T cells, especially in terms of chronic antigen exposure at LIT.

Cbl-b^−/−^ mice in our model exhibited a modulation in the CD4^+^ CD25^+^ Foxp3^−^ T cell population during AAD as these cells were increased as a percentage of the total CD4^+^ T cell population in the BAL as compared to wild-type BAL. This increase in Foxp3^−^ T cells in local lung compartments during AAD may be influencing the increase in airway inflammation seen in Cbl-b^−/−^ mice. At LIT, wild-type and Cbl-b^−/−^ mice had equivalent frequencies of Foxp3^−^ and Foxp3^+^ T cells in the BAL, which appears in contrast to the increased airway inflammation seen at LIT in Cbl-b^−/−^ mice. However, the inability to resolve airway inflammation in Cbl-b^−/−^ mice at LIT may be due to the “resistant to regulation” phenotype of Cbl-b^−/−^ T cells and not to enhanced Foxp3^−^ T cell populations as seen at AAD. These results suggest that separate mechanisms may control the severity of inflammation at AAD and LIT, with both involving Cbl-b. For example, the inability of Cbl-b^−/−^ T cells to be regulated by Foxp3^+^ T_reg_ may play a role in the expanded Foxp3^−^ activated T cell pool seen at AAD, while a combination of resistance to “(extrinsic) regulation” by T_reg_ and “(intrinsic) regulation” through anergy induction may result in the maintenance of airway inflammation at LIT. Further studies are required to assess the functionality of T cell subpopulations in the BAL at AAD and LIT in Cbl-b mice, along with the role of TGF-β, which has been shown to be induced as a result of OVA aerosol challenge in this model (17).

For the most part, the effect of Cbl-b deficiency on the severity of inflammation during AAD and LIT did not result in modulations in lymphocyte populations. For example, Cbl-b^−/−^ mice did not exhibit increases in CD4^+^ or CD8^+^ T cells, or CD19^+^ B cells, in local lung or systemic compartments during AAD and LIT, suggesting that the exacerbation of inflammation in Cbl-b^−/−^ mice was not due to increases in numbers of effector lymphocytes. Interestingly, a general increase of total numbers of all lymphocytes analyzed was observed in the HLN of Cbl-b^−/−^ mice at LIT. Previous studies have indicated that T cells lacking Cbl-b exhibit a hyperproliferative phenotype *in vitro* (5, 9), suggesting a possible cause for this observed expansion of T cells in this *in vivo* model. Of additional interest is the skewing of the T cell response in Cbl-b^−/−^ mice toward CD8^+^ T cells, especially in the BAL. Cbl-b^−/−^ mice exhibited a decreased CD4:CD8 T cell ratio at both AAD and LIT as compared to wild-type mice, which is unexpected due to the T_H_2-type nature of the OVA-induced AAD response. Little is known about the role of Cbl-b in T-helper polarization; however, Cbl-b has been indicated in TCR-mediated activation-induced cell death (AICD) in T_H_1 cells (34), and has been shown to play a role in skewing thymocyte development toward CD8^+^ T cells in c-Cbl/Cbl-b dual^−/−^ mice (35). These studies provide evidence that dysregulated T_H_1 responses or CD8^+^ T cell development in Cbl-b^−/−^ mice could result in the increase in CD8^+^ T cells at AAD and LIT.

In addition, Cbl-b has been shown to play an important role in negatively regulating B cell receptor (36) and CD40 signal strength (37), suggesting a similar mechanism governing the expansion of B cells in the HLN of Cbl-b^−/−^ mice during LIT. However, conflicting reports suggest that Cbl-b acts as a positive regulator of B cell activation through interactions with TRANCE receptor (38) and Erk (39) with downstream effects on calcium mobilization and the signal transduction proteins Akt and Lyn. While the expansion of B cells in the HLN during LIT appears to correlate with increased inflammation in the lungs of Cbl-b^−/−^ mice, it is not cell-specific, as all lymphocyte subsets analyzed were increased in the HLN of Cbl-b^−/−^ mice at LIT. Similarly, while serum IgE levels were slightly elevated in Cbl-b^−/−^ mice as compared to wild-type controls, they were not statistically significant. This observation may be explained by the observed patterns of cytokine expression observed in this animal model. For example, despite elevated production of specific T_H_2 cytokines in Cbl-b^−/−^ mice, such as IL-5 and IL-13, there was no significant elevation in IL-4 production observed. As IL-4 is the primary T_H_2 cytokine responsible for IgE class-switching (40), the equivalent production of IgE in Cbl-b^−/−^ mice during AAD and LIT may be due to the equivalent IL-4 production observed concurrently at both timepoints. Regardless, these findings suggest a possible role for B cell dysregulation in Cbl-b^−/−^ mice in this model, albeit one that needs to be further elucidated.

Previous work indicates that OVA-induced allergic airway inflammation in mice can be induced in B cell deficient mice (41, 42). However, recent studies have indicated that Cbl-b plays an important role in regulating mast cell activation (43) and subsequent cytokine production (44) by IgE-activated mast cells, which are critical mediators in allergic lung inflammation in both mice (45, 46) and humans (47, 48). Therefore, exacerbation of disease in Cbl-b^−/−^ mice may be due to a combination of B cell and mast cell dysregulation, correlated with IgE crosslinking of FcϵRI during aerosol challenge. As discussed previously, Cbl-b has also been shown to play important roles in mediating anergy induction in other lymphoid subsets in addition to B cells and mast cells, such as NK and NK-T cells. In the experimental animal model utilized in this study (Cbl-b^−/−^), all leukocyte subsets are deficient in Cbl-b expression, meaning that there is probably a pleiotropic effect of Cbl-b deficiency on global cellular immune responses in this inhaled allergy model. Further studies are required to assess the contribution of Cbl-b deficiency on the function of other immune cell subsets in the context of OVA-induced AAD and LIT.

In conclusion, Cbl-b^−/−^ mice exhibit increased inflammatory responses to OVA-induced allergic airway inflammation, including exacerbations in disease outcomes at both AAD and LIT timepoints, and increases in T cell chemokine and proinflammatory T_H_2 cytokines in the BAL. Conversely, Cbl-b^−/−^ mice did not show modulations in T or B cells in local or systemic compartments during AAD or LIT, nor was there a significant modulation in serum IgE production at either timepoint. Finally, Cbl-b did exhibit a increase in a possibly polyclonal cell population with an effector T cell phenotype in the BAL during AAD, suggesting a dysregulation of T cell responses during acute disease. These findings indicate a role for Cbl-b in the regulation and resolution of allergic airway responses, along with suggesting a correlation between disease severity and impaired regulatory T cell functions.

## Author Contributions

All authors assisted in data collection and analysis, and assisted in manuscript preparation and revision. WC, CS, RC, and RT designed the experiments. RC, CS, SK, and RT provided reagents, equipment, and experimental animals. WC, LG, AS, ES, and EW performed the experiments. WC, CS, and RT wrote the manuscript.

## Conflict of Interest Statement

The authors declare that the research was conducted in the absence of any commercial or financial relationships that could be construed as a potential conflict of interest.

## References

[1] Appleman LJ Boussiotis VA. T cell anergy and costimulation. Immunol Rev (2003) 192:161–80. 10.1034/j.1600-065X.2003.00009.x 12670403

[2] Schwartz RH. T cell anergy. Annu Rev Immunol (2003) 21:305–34. 10.1146/annurev.immunol.21.120601.141110 12471050

[3] Liu YC Gu H. Cbl and Cbl-b in T-cell regulation. Trends Immunol (2002) 23:140–3. 10.1016/S1471-4906(01)02157-3 11864842

[4] Thien CB Langdon WY. c-Cbl and Cbl-b ubiquitin ligases: substrate diversity and the negative regulation of signalling responses. Biochem J (2005) 391:153–66. 10.1042/BJ20050892 16212556 PMC1276912

[5] Chiang YJ Kole HK Brown K Naramura M Fukuhara S Hu RJ Cbl-b regulates the CD28 dependence of T-cell activation. Nature (2000) 403:216–20. 10.1038/35003235 10646609

[6] Naramura M Jang IK Kole H Huang F Haines D Gu H. c-Cbl and Cbl-b regulate T cell responsiveness by promoting ligand-induced TCR down-modulation. Nat Immunol (2002) 3:1192–9. 10.1038/ni855 12415267

[7] Heissmeyer V Macian F Im SH Varma R Feske S Venuprasad K Calcineurin imposes T cell unresponsiveness through targeted proteolysis of signaling proteins. Nat Immunol (2004) 5:255–65. 10.1038/ni1047 14973438

[8] Qiao G Li Z Molinero L Alegre ML Ying H Sun Z T-cell receptor-induced NF-kappaB activation is negatively regulated by E3 ubiquitin ligase Cbl-b. Mol Cell Biol (2008) 28:2470–80. 10.1128/MCB.01505-07 18227156 PMC2268433

[9] Bachmaier K Krawczyk C Kozieradzki I Kong YY Sasaki T Oliveira-Dos-Santos A Negative regulation of lymphocyte activation and autoimmunity by the molecular adaptor Cbl-b. Nature (2000) 403:211–6. 10.1038/35003228 10646608

[10] Paolino M Choidas A Wallner S Pranjic B Uribesalgo I Loeser S The E3 ligase Cbl-b and TAM receptors regulate cancer metastasis via natural killer cells. Nature (2014) 507:508–12. 10.1038/nature12998 24553136 PMC6258903

[11] Kojo S Elly C Harada Y Langdon WY Kronenberg M Liu YC. Mechanisms of NKT cell anergy induction involve Cbl-b-promoted monoubiquitination of CARMA1. Proc Natl Acad Sci U S A (2009) 106:17847–51. 10.1073/pnas.0904078106 19815501 PMC2764888

[12] Kitaura Y Jang IK Wang Y Han YC Inazu T Cadera EJ Control of the B cell-intrinsic tolerance programs by ubiquitin ligases Cbl and Cbl-b. Immunity (2007) 26:567–78. 10.1016/j.immuni.2007.03.015 17493844 PMC1948079

[13] Zhang J Chiang YJ Hodes RJ Siraganian RP. Inactivation of c-Cbl or Cbl-b differentially affects signaling from the high affinity IgE receptor. J Immunol (2004) 173:1811–8. 10.4049/jimmunol.173.3.1811 15265912

[14] Krawczyk CM Jones RG Atfield A Bachmaier K Arya S Odermatt B Differential control of CD28-regulated in vivo immunity by the E3 ligase Cbl-b. J Immunol (2005) 174:1472–8. 10.4049/jimmunol.174.3.1472 15661906

[15] Wohlfert EA Callahan MK Clark RB. Resistance to CD4+CD25+ regulatory T cells and TGF-beta in Cbl-b-/- mice. J Immunol (2004) 173:1059–65. 10.4049/jimmunol.173.2.1059 15240694

[16] Wohlfert EA Gorelik L Mittler R Flavell RA Clark RB. Cutting edge: deficiency in the E3 ubiquitin ligase Cbl-b results in a multifunctional defect in T cell TGF-beta sensitivity in vitro and in vivo. J Immunol (2006) 176:1316–20. 10.4049/jimmunol.176.3.1316 16424156

[17] Schramm CM Puddington L Wu C Guernsey L Gharaee-Kermani M Phan SH Chronic inhaled ovalbumin exposure induces antigen-dependent but not antigen-specific inhalational tolerance in a murine model of allergic airway disease. Am J Pathol (2004) 164:295–304. 10.1016/S0002-9440(10)63119-7 14695342 PMC1602237

[18] Gavett SH Chen X Finkelman F Wills-Karp M. Depletion of murine CD4+ T lymphocytes prevents antigen-induced airway hyperreactivity and pulmonary eosinophilia. Am J Respir Cell Mol Biol (1994) 10:587–93. 10.1165/ajrcmb.10.6.8003337 8003337

[19] Carson WF IV Guernsey LA Singh A Vella AT Schramm CM Thrall RS. Accumulation of regulatory T cells in local draining lymph nodes of the lung correlates with spontaneous resolution of chronic asthma in a murine model. Int Arch Allergy Immunol (2008) 145:231–43. 10.1159/000109292 17914275 PMC2576511

[20] Qiao G Ying H Zhao Y Liang Y Guo H Shen H E3 ubiquitin ligase Cbl-b suppresses proallergic T cell development and allergic airway inflammation. Cell Rep (2014) 6:709–23. 10.1016/j.celrep.2014.01.012 24508458 PMC3969736

[21] Yiamouyiannis CA Schramm CM Puddington L Stengel P Baradaran-Hosseini E Wolyniec WW Shifts in lung lymphocyte profiles correlate with the sequential development of acute allergic and chronic tolerant stages in a murine asthma model. Am J Pathol (1999) 154:1911–21. 10.1016/S0002-9440(10)65449-1 10362818 PMC1866641

[22] Renz H Smith HR Henson JE Ray BS Irvin CG Gelfand EW. Aerosolized antigen exposure without adjuvant causes increased IgE production and increased airway responsiveness in the mouse. J Allergy Clin Immunol (1992) 89:1127–38. 10.1016/0091-6749(92)90296-E 1607548

[23] Hamelmann E Gelfand EW IL-5-induced airway eosinophilia – the key to asthma? Immunol Rev (2001) 179:182–91. 10.1034/j.1600-065X.2001.790118.x 11292022

[24] Ngoc PL Gold DR Tzianabos AO Weiss ST Celedon JC Cytokines, allergy, and asthma. Curr Opin Allergy Clin Immunol (2005) 5:161–6. 10.1097/01.all.0000162309.97480.45 15764907

[25] Broide DH. Immunologic and inflammatory mechanisms that drive asthma progression to remodeling. J Allergy Clin Immunol (2008) 121:560–70; quiz 571-562. 10.1016/j.jaci.2008.01.031 18328887 PMC2386668

[26] Doganci A Sauer K Karwot R Finotto S Pathological role of IL-6 in the experimental allergic bronchial asthma in mice. Clin Rev Allergy Immunol (2005) 28:257–70. 10.1385/CRIAI:28:3:257 16129910

[27] Doganci A Eigenbrod T Krug N De Sanctis GT Hausding M Erpenbeck VJ The IL-6R alpha chain controls lung CD4+CD25+ Treg development and function during allergic airway inflammation in vivo. J Clin Invest (2005) 115:313–25. 10.1172/JCI22433C1 15668741 PMC544603

[28] Wan S Xia C Morel L. IL-6 produced by dendritic cells from lupus-prone mice inhibits CD4+CD25+ T cell regulatory functions. J Immunol (2007) 178:271–9. 10.4049/jimmunol.178.1.271 17182564

[29] Rothenberg ME Hogan SP. The eosinophil. Annu Rev Immunol (2006) 24:147–74. 10.1146/annurev.immunol.24.021605.090720 16551246

[30] Takatsu K Nakajima H. IL-5 and eosinophilia. Curr Opin Immunol (2008) 20:288–94. 10.1016/j.coi.2008.04.001 18511250

[31] Heissmeyer V Rao A E3 ligases in T cell anergy – turning immune responses into tolerance. Sci STKE (2004) 2004:e29 10.1126/stke.2412004pe29 15252218

[32] Mueller DL. E3 ubiquitin ligases as T cell anergy factors. Nat Immunol (2004) 5:883–90. 10.1038/ni1106 15334084

[33] Jeon MS Atfield A Venuprasad K Krawczyk C Sarao R Elly C Essential role of the E3 ubiquitin ligase Cbl-b in T cell anergy induction. Immunity (2004) 21:167–77. 10.1016/j.immuni.2004.07.013 15308098

[34] Hanlon A Jang S Salgame P. Cbl-b differentially regulates activation-induced apoptosis in T helper 1 and T helper 2 cells. Immunology (2005) 116:507–12. 10.1111/j.1365-2567.2005.02252.x 16313364 PMC1802430

[35] Huang F Kitaura Y Jang I Naramura M Kole HH Liu L Establishment of the major compatibility complex-dependent development of CD4+ and CD8+ T cells by the Cbl family proteins. Immunity (2006) 25:571–81. 10.1016/j.immuni.2006.08.021 17045823

[36] Sohn HW Gu H Pierce SK. Cbl-b negatively regulates B cell antigen receptor signaling in mature B cells through ubiquitination of the tyrosine kinase Syk. J Exp Med (2003) 197:1511–24. 10.1084/jem.20021686 12771181 PMC2193911

[37] Qiao G Lei M Li Z Sun Y Minto A Fu YX Negative regulation of CD40-mediated B cell responses by E3 ubiquitin ligase Casitas-B-lineage lymphoma protein-B. J Immunol (2007) 179:4473–9. 10.4049/jimmunol.179.7.4473 17878343

[38] Arron JR Vologodskaia M Wong BR Naramura M Kim N Gu H A positive regulatory role for Cbl family proteins in tumor necrosis factor-related activation-induced cytokine (trance) and CD40L-mediated Akt activation. J Biol Chem (2001) 276:30011–7. 10.1074/jbc.M100414200 11406619

[39] Shao Y Yang C Elly C Liu YC. Differential regulation of the B cell receptor-mediated signaling by the E3 ubiquitin ligase Cbl. J Biol Chem (2004) 279:43646–53. 10.1074/jbc.M404082200 15304502

[40] Kuhn R Rajewsky K Muller W. Generation and analysis of interleukin-4 deficient mice. Science (1991) 254:707–10. 10.1126/science.1948049 1948049

[41] Hamelmann E Takeda K Schwarze J Vella AT Irvin CG Gelfand EW. Development of eosinophilic airway inflammation and airway hyperresponsiveness requires interleukin-5 but not immunoglobulin E or B lymphocytes. Am J Respir Cell Mol Biol (1999) 21:480–9. 10.1165/ajrcmb.21.4.3659 10502558

[42] Singh A Carson WF IV Secor ER Jr Guernsey LA Flavell RA Clark RB Regulatory role of B cells in a murine model of allergic airway disease. J Immunol (2008) 180:7318–26. 10.4049/jimmunol.180.11.7318 18490731 PMC2576522

[43] Qu X Sada K Kyo S Maeno K Miah SM Yamamura H. Negative regulation of FcepsilonRI-mediated mast cell activation by a ubiquitin-protein ligase Cbl-b. Blood (2004) 103:1779–86. 10.1182/blood-2003-07-2260 14604964

[44] Gustin SE Thien CB Langdon WY. Cbl-b is a negative regulator of inflammatory cytokines produced by IgE-activated mast cells. J Immunol (2006) 177:5980–9. 10.4049/jimmunol.177.9.5980 17056522

[45] Taube C Wei X Swasey CH Joetham A Zarini S Lively T Mast cells, Fc epsilon RI, and IL-13 are required for development of airway hyperresponsiveness after aerosolized allergen exposure in the absence of adjuvant. J Immunol (2004) 172:6398–406. 10.4049/jimmunol.172.10.6398 15128831

[46] Yu M Tsai M Tam SY Jones C Zehnder J Galli SJ. Mast cells can promote the development of multiple features of chronic asthma in mice. J Clin Invest (2006) 116:1633–41. 10.1172/JCI25702 16710480 PMC1462940

[47] Bradding P Walls AF Holgate ST. The role of the mast cell in the pathophysiology of asthma. J Allergy Clin Immunol (2006) 117:1277–84. 10.1016/j.jaci.2006.02.039 16750987

[48] Krishnaswamy G Ajitawi O Chi DS. The human mast cell: an overview. Methods Mol Biol (2006) 315:13–34. 10.1385/1-59259-967-2:013 16110146

